# Evaluating the influence of novel charge transport materials on the photovoltaic properties of MASnI_3_ solar cells through SCAPS-1D modelling

**DOI:** 10.1098/rsos.231202

**Published:** 2024-01-17

**Authors:** Khalid Afridi, Muhammad Noman, Shayan Tariq Jan

**Affiliations:** ^1^ U.S.-Pakistan Center for Advanced Studies in Energy, University of Engineering and Technology, Peshawar 25000, Pakistan; ^2^ Department of Energy Engineering Technology, University of Technology, Nowshera, Pakistan

**Keywords:** perovskite solar cell, MASnI_3_, carbon ETL, copper HTL, SCAPS-1D

## Abstract

In recent decades, substantial advancements have been made in photovoltaic technologies, leading to impressive power conversion efficiencies (PCE) exceeding 25% in perovskite solar cells (PSCs). Tin-based perovskite materials, characterized by their low band gap (1.3 eV), exceptional optical absorption and high carrier mobility, have emerged as promising absorber layers in PSCs. Achieving high performance and stability in PSCs critically depends on the careful selection of suitable charge transport layers (CTLs). This research investigates the effects of five copper-based hole transport materials and two carbon-based electron transport materials in combination with methyl ammonium tin iodide (MASnI_3_) through numerical modelling in SCAPS-1D. The carbon-based CTLs exhibit excellent thermal conductivity and mechanical strength, while the copper-based CTLs demonstrate high electrical conductivity. The study comprehensively analyses the influence of these CTLs on PSC performance, including band alignment, quantum efficiency, thickness, doping concentration, defects and thermal stability. Furthermore, a comparative analysis is conducted on PSC structures employing both p-i-n and n-i-p configurations. The highest-performing PSCs are observed in the inverted structures of CuSCN/MASnI_3_/C_60_ and CuAlO_2_/MASnI_3_/C_60_, achieving PCE of 23.48% and 25.18%, respectively. Notably, the planar structures of Cu_2_O/MASnI_3_/C_60_ and CuSbS_2_/MASnI_3_/C_60_ also exhibit substantial PCE, reaching 20.67% and 20.70%, respectively.

## 1. Introduction

Renewable energy sources have become a crucial component for the development and sustainability of human society, with solar energy being the primary contender [1]. Perovskite solar cells (PSCs) have established their superiority over silicon photovoltaic cells owing to their simplicity and affordability of fabrication through wet chemistry techniques [2]. The third-generation thin-film organic-metal halide has generated immense attention from the research community owing to its notable efficiency, cost-effectiveness and potential to satisfy the escalating need for clean energy [3–5].

The absorber layer of PSC has a general stoichiometry of ABX_3_ [6,7], where ‘A’ represents an organic cation (methyl ammonium-MA, formamidinium-FA, cesium-Ce) [8], ‘B’ represents a metal ion (lead-Pb, tin-Sn, germanium-Ge) and ‘X’ represent halide ions (Cl, Br and I) [9]. Methyl ammonium lead iodide (MAPbI_3_) is the most commonly used perovskite material as an absorber layer in photovoltaic cells. The development of technology has resulted in a remarkable increase in the power conversion efficiency (PCE) of PSCs, from 3.8% to 25.7%. This notable improvement has positioned PSCs as a viable alternative to conventional photovoltaic cells. [3,10].

The major hurdle in PSC commercialization is the toxic nature of lead in MAPbI_3_ [11,12]. Extensive theoretical and experimental studies have been carried out to identify alternative high-performing non-toxic perovskite materials [13]. Non-toxic elements such as Sn, Ge, Cu and Bi have been investigated and identified as a replacement for lead [14]. Out of these, Sn is one of the most promising substitutes for Pb because of its similar optoelectronic properties [15,16] Tin(Sn)-based PSC has better optoelectronic properties such as long diffusion length and higher absorption [5,17] owing to its direct and narrower band gap of 1.3 eV [18]. These properties give Sn-based PSC the potential to achieve higher PCE than its other counterparts [19,20].

Methyl ammonium tin iodide (MASnI_3_) is characterized by its outstanding stability, an essential feature for its use in photovoltaic devices that demand long-term performance [21]. The materials naturally have low ionization potential, which plays a pivotal role in ensuring the stability of both tin vacancy and interstitial iodine defects [22]. The defects within MASnI_3_ actively contribute to the p-doping of the material, thereby enhancing its electronic properties and improving its resilience against environmental elements [23]. The hexagonal phase of MASnI_3_ boasts an indirect bandgap and a more significant carrier-effective mass along the *c*-axis than its cubic and tetragonal counterparts [24]. This distinctive characteristic of the material allows the absorbtion of ultraviolet photons effectively, interpreting it flexible for various applications [25]. Notably, MASnI_3_ composition is lead-free, presenting an eco-friendly alternative to other perovskites [26]. Devices created from MASnI_3_, particularly those that are Sn-based hollow perovskites, demonstrate superior air stability, offering a dependable solution for crafting efficient and sustainable lead-free solar cells [27]. The calculated theoretical maximum PCE for MASnX_3_, FASnX_3_ and CsSnI_3_-based PSCs stands at 19.9%, 26.9% and 25.6%, respectively [28]. Initially, the PCE of these cells was around 5.73% with a limited shelf life of 12 h. However, through further research and development, the PCE has now exceeded 13% and the stability has reached 3800 h [29].).

The selection of suitable charge transport layers (CTL) for the PSC structures is of utter importance to achieve improved efficiency and stability [1]. Organic hole transport layers (HTLs) inherently exhibit lower electrical conductivity compared to inorganic HTLs, limiting the efficient transport of charge carriers and potentially diminishing the overall efficiency of PSCs [30]. To address the low conductivity issue, one might dope organic HTLs with additional materials [31]. This doping process introduces extra charge carriers, thereby enhancing the conductivity within the organic layer [32].

By contrast, inorganic HTLs naturally possess higher electrical conductivity, ensuring efficient charge transport [33]. This higher conductivity is vital for PSCs' effective operation, allowing charge carriers to move rapidly and leading to increased PCEs [34].

Environmental factors like moisture, oxygen and temperature often affect organic materials, leading to their degradation over time [35]. This degradation process can impact the PSCs’ performance and longevity adversely [36]. On the other hand, inorganic HTLs exhibit greater stability and resistance to environmental degradation, making them a reliable choice for long-term applications [37].

Notably, inorganic HTLs stand out for their robustness and durability [38]. They offer enhanced mechanical strength and resistance to physical degradation, making them ideal for applications that demand resilient and durable materials [39]. Furthermore, inorganic HTLs showcase superior thermal stability, maintaining their performance and integrity even under elevated temperatures [40]. This feature makes them particularly suitable for applications where solar cells face exposure to high temperatures [41].

Compatibility with a wide range of perovskite materials is another advantage of inorganic HTLs [42]. This compatibility allows for the creation of diverse combinations of materials to achieve the desired photovoltaic properties for various applications [43]. Moreover, inorganic HTLs effectively resist chemical and environmental degradation, ensuring PSCs maintain sustained performance and longevity, even under challenging environmental conditions [44].

With enhanced electrical and thermal properties, inorganic HTLs play a crucial role in achieving higher PCEs in PSCs, resulting in superior performance [45]. Overall, when considering factors like conductivity, stability, durability, thermal stability, compatibility, environmental resistance, performance and technology maturity, inorganic HTLs hold distinct advantages over organic HTLs in the realm of PSCs [46].

TiO_2_ material has been found to be a suitable electron transport layer (ETL) for the PSC but it faces the issue of degradation owing to the presence of oxygen vacancies [47]. Similarly, Spiro-OMeTAD has been found to be the most extensively used HTL but with high material cost [48,49]. This has led researchers to explore alternate high-performing CTLs. Studies have shown that there are multiple options to choose from for the CTL but not all perform at the same level owing to the difference in band offset, hetero-junction electric field and carrier mobility. Identifying the best CTL is a vital part of making the PSC structure [50]. There are various organic–inorganic materials for HTL and ETL to be chosen from [51]. The low efficiency of the perovskite device can be diminished by using alternative HTL materials such as CuI [52], Cu_2_O, CuSbS_2_, CuSCN and CuAlO_2_ [53] with lead-free perovskite. The Cu-based materials have high electric conductivity and when used in the PSC, have shown to enhance the performance of the cell [21,54–56]. Similarly, studies have shown that using alternative carbon-based materials such as fullerene derivative [6,6]-phenyl-C61-butyric acid methyl ester (PCBM) and C_60_ not only improves the mechanical strength of the cell but also its thermal conductivity ([57,58], owing to their low hysteresis [59–62].

In this work, after carrying out an extensive study of the literature, five Cu-based materials (CuI, Cu_2_O, CuSCN, CuAlO_2_ and CuSbS_2_) and two carbon-based materials (PCBM and C_60_) have been identified to be used with MASnI_3_ to boost its performance. A total of 10 PSC structures with different CTL combinations have been modelled in SCAPS-1D and studied in detail. The influence of CTL on the band alignment, thickness, quantum efficiency (QE), doping concentration, defect density, temperature stability, interface defect and work function has been carried out.

## 2. Device structure modelling

SCAPS-1D software is used for modelling and optimization of the PSC device parameter. The most important parameters such as thickness, doping density and defect density of absorber layers are focussed on which can significantly affect the performance of the PSC. In this simulation study, MASnI_3_ is analysed as a light-absorbing layer with different CTLs, while carbon-based ETL is selected to enhance the thermal conductivity and mechanical strength of the PSC. The structure of anode/HTL/MASnI_3_/ETL/cathode is modelled for all the PSC structures in SCAPS. Owing to the low band gap of carbon, inverted p-i-n structures are modelled. For the HTL, copper-based materials are focussed on owing to their larger band gap and high electrical conductivity. The structures are then also analysed in planner n-i-p arrangement and the performance of both planar and inverted are compared. The regular planar perovskite device of the n-i-p is shown in the electronic supplementary material, figure S1 and the p-i-n inverted structure is shown in the electronic supplementary material, figure S2. Fluorine-doped tin iodide (FTO) is used as front contact and platinum (Pt) is used as a back electrode in n-i-p structure. While in the inverted perovskite, indium-tin oxide (ITO) is used as the front electrode, and aluminium (Al) is used as the back contact. In addition, the bulk defects in all the layers and the interface defects (HTL/perovskite and ETL/perovskite) are also modelled in all the structures to get more practical results. The energy level diagram (electronic supplementary material, figure S3) demonstrates the e-h pair generated by the absorber layer from the absorbed photon. The electrons flow through the conduction band (CB) to ETL and holes to the HTL through the valance band (VB). The primary causes of interface defects are dangling bonds, surface imperfections and grain boundaries between the two layers. The design parameters used for the interface defect are shown in the electronic supplementary material, table S3.

The various parameters such as band gap, CB/VB effective state of density, electron affinity, dielectric permittivity, electron/hole mobility, thermal velocity and doping density have been derived from various research papers and their references have been cited in the electronic supplementary material, tables S1 and S2.

The calculations in this study are based on the fundamental principles of semi-conductors which include the Poisson equations (equation (2.1)) and the continuity equation (equations (2.2) and (2.3)):

2.1 $$\frac{d}{dx}\left[\varepsilon (x)\frac{d\psi }{dx}\right]=q[p(x)-n(x)-N_{D}(x)-N_{A}(x)-n_{t}(x)+p_{t}(x),$$

2.2 $$-\frac{1}{q}\frac{d}{d}\frac{J_{n}}{x}\;+R_{n}(x)-G(x)=0$$

2.3 $$\mathrm{and}\;\;\;\;\;\;\;\;\frac{1}{q}\frac{d}{d}\frac{J_{p}}{x}\;+R_{p}(x)-G(x)=0,$$

where *N_A_* and *N_D_* are the concentration of the acceptor and donor; *x* indicates the coordinate position; symbol *ψ* denotes electrostatic potential; *p* and *n* represent the number of holes and electrons; *p_t_* and *n_ot_* specify the number of trap holes and electrons; *J_p_* and *J_n_* indicate the current density of holes and electrons; *G*(*x*) is the optical generation; and *R_p_*(*x*) and *R_n_*(*x*) entitle the rate of recombination of holes and electrons.

The relationship between the drift and diffusion for electron and hole is given by the equation of continuity:

2.4 $$J_{n}=qn\mu_{n}E+qD_{n}\nabla n$$

and

2.5 $$J_{p}=qp\mu_{p}E+qD_{p}\nabla n,$$

where *D_n_* is the diffusion coefficient for electrons; *D_p_* is the diffusion coefficient for holes.

## 3. Results and discussion

Different combinations of electron and hole transport materials with the absorber layer of MASnI_3_ have been modelled to study the effect of various parameters in SCAPS-1D. Several PSC device configurations have been analysed as inverted structures owing to the high transparency of hole transfer materials.

### 3.1. Absorption

**Figure 1.**
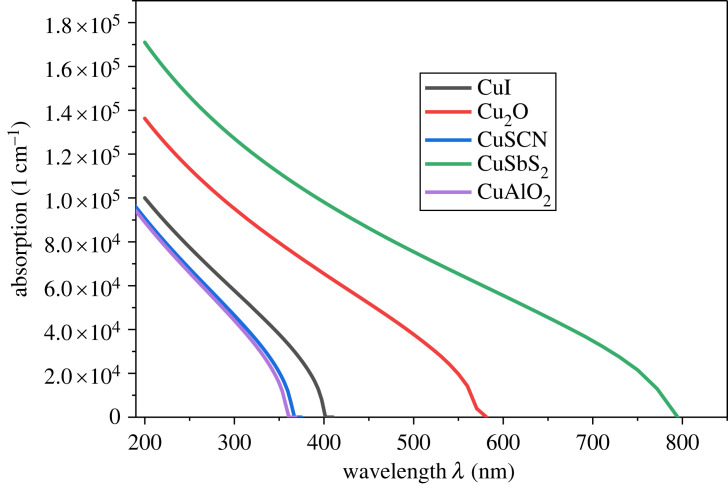
Optical absorption of HTL.

Materials with high absorption coefficients can absorb a large fraction of incident radiation, while materials with a low absorption coefficient are less effective at absorbing radiation [63]. The absorbance of CTL depends on specific parameters such as the wavelength of the incident radiation and the physical and chemical properties of CTL [64]. The absorption of the mentioned inorganic p-type HTLs is shown in figure 1. It is observed that the absorption of HTL CuSbS_2_ is high and can absorb photons of a high wavelength (up to 800 nm) owing to its narrow bandgap. Therefore, the transparency of copper antimony sulfide is low. The HTLs such as CuAlO_2_ and CuSCN have shown low absorption and high transmissivity because of the small absorption coefficient and wider bandgap.

**Figure 2.**
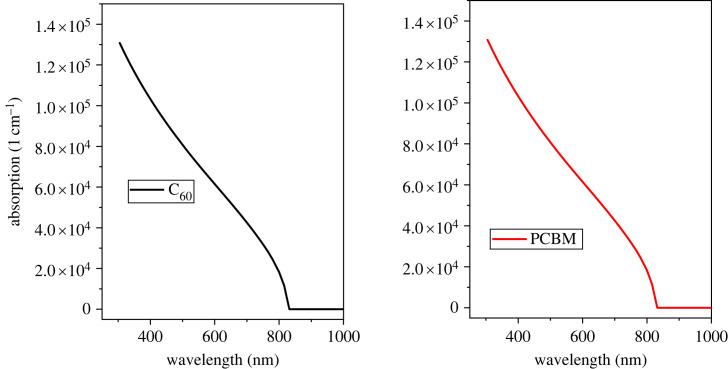
Optical absorption of ETL.

The absorption coefficient of the ETL is higher than HTL and has low transparency as observed in figure 2. In terms of optical absorbance, C_60_ and PCBM exhibit strong absorption in the visible and near-infrared regions of the electromagnetic spectrum.

## 4. Energy band alignment

The band offsets (conduction band offset (CBO) and valance band offset (VBO)) are an important parameter of the thin film PSC [65]. The difference between the CB of CTL and that of the absorber is known as CBO, while VBO is defined as the difference between the VB of the CTL and perovskite [66].

**Figure 3.**
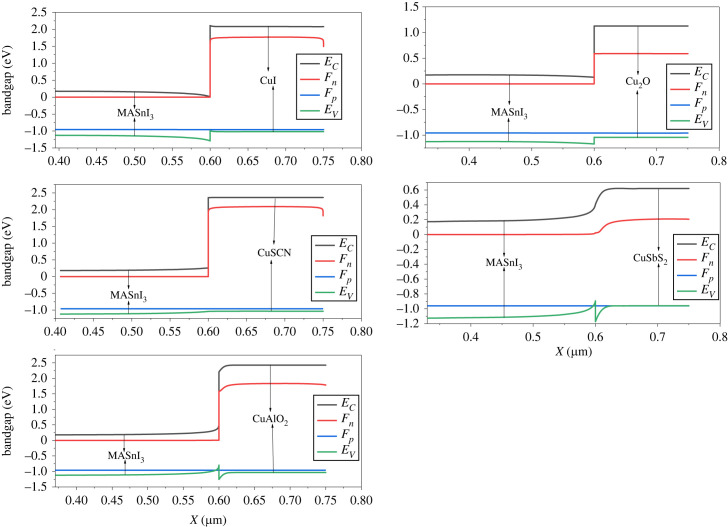
The energy band diagram of the perovskite/HTL system.

The highest occupied molecular orbital (HOMO) of the active layer should be slightly lower than the HTL for the holes to be extracted from the absorber layer to the HTL. In addition to this, it is preferred that the perovskite lowest energy orbital (LUMO) has a large gap with the HTL to block the flow of electrons to the HTL [67]. The adjustment of the VBO and CBO is a critical issue for the efficient extraction of holes from the perovskite to HTL [51,68]. The band alignment of various hole transport materials is shown in figure 3 where it can be observed that the inorganic copper-based HTL has appropriate band alignment. The VBO of the HTLs CuSCN and CuAlO_2_ is 0 and 0.46 eV, while its CBO is 2.1 and 1.7 eV (table 1). Owing to the high CBO at PAL/CuSCN and PAL/CuAlO_2_, they have minimum leakage of electrons to the HTL, therefore showing high performance. The CBO of CuSbS_2_ is small (0.2 eV), which causes leakage of electrons to the HTL, leading to recombination and poor performance.

**Table 1.** Band offset values of HTL.

HTL	VBO_HTL_ (eV)	CBO_HTL_ (eV)
CuI	−0.3	2.1
Cu_2_O	−0.13	1
CuSCN	0	2.1
CuSbS_2_	0.28	0.2
CuAlO_2_	0.46	1.7

**Figure 4.**
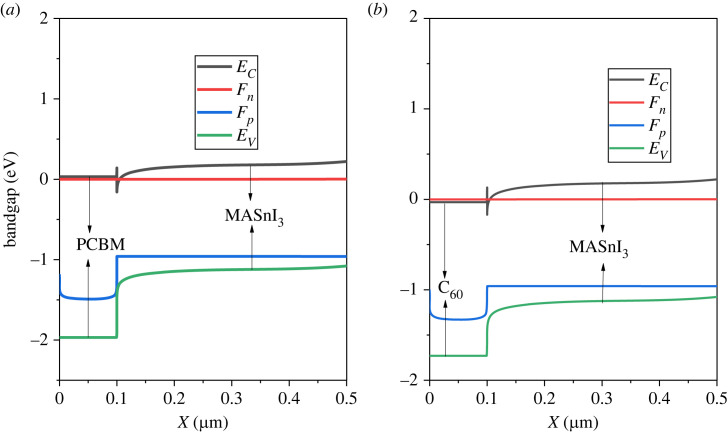
The energy band diagram of the ETL/perovskite system.

The band diagram of the ETL is shown in figure 4. For the ETL, the CBO should be minimum with the perovskite so that electrons can flow to the ETL, while the VBO should be maximum to reduce the leakage of holes. The HOMO and LUMO level of the PCBM as the ETL matched well with the perovskite, therefore a good transport of the electrons and blocking of the holes from the active layer occurred at the interface absorber/ETL. The CBO and VBO of the ETL are given in table 2.

**Table 2.** Band offset values of ETL.

ETL	VBO_ETL_ (eV)	CBO_ETL_ (eV)
PCBM	0.4	0.3
C_60_	0.1	0.3

### 4.1. Electric potential

**Figure 5.**
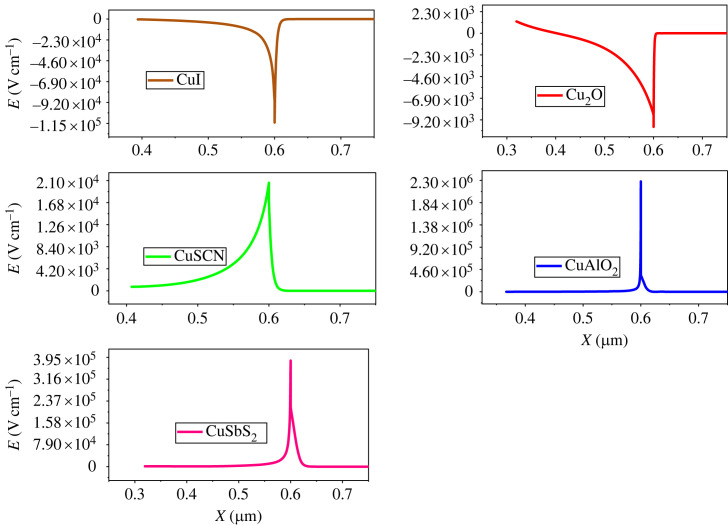
Electric potential of different hole transport materials with perovskite.

**Figure 6.**
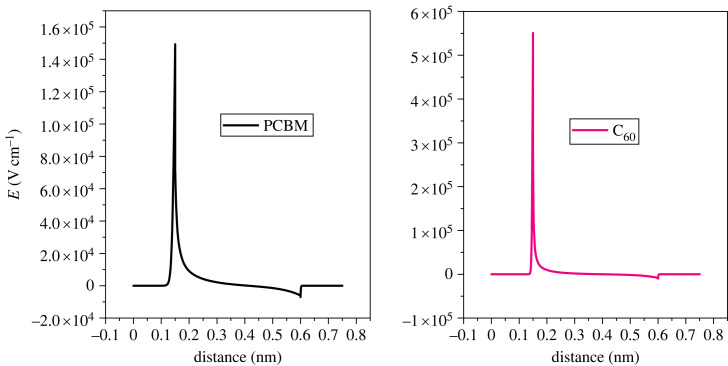
Electric potential of different electron transport materials with perovskite.

The charger carriers from the absorber layer are separated with the help of an electric field at the heterojunction [69]. Figure 5 depicts the electric field observed at the interface between the HTL and perovskite, while figure 6 illustrates the electric field at the interface between the perovskite and ETL. The alignment of the band structure exerts a notable influence on the electric field generated at the interface between the absorber and the CTL. If the CTL and perovskite make a positive band offset, a spike is formed which increases the built-in potential of the cell. However, if the band offset is negative, a cliff is formed which reduces the potential. The electric field potential of HTL (CuSCN, CuAlO_2_ and CuSbS_2_) is high owing to the spike at the heterojunction, while the electrical potential of Cu_2_O and CuI is low owing to the cliff interfaces.

### 4.2. Recombination at charge transport layers/perovskite heterojunction

**Figure 7.**
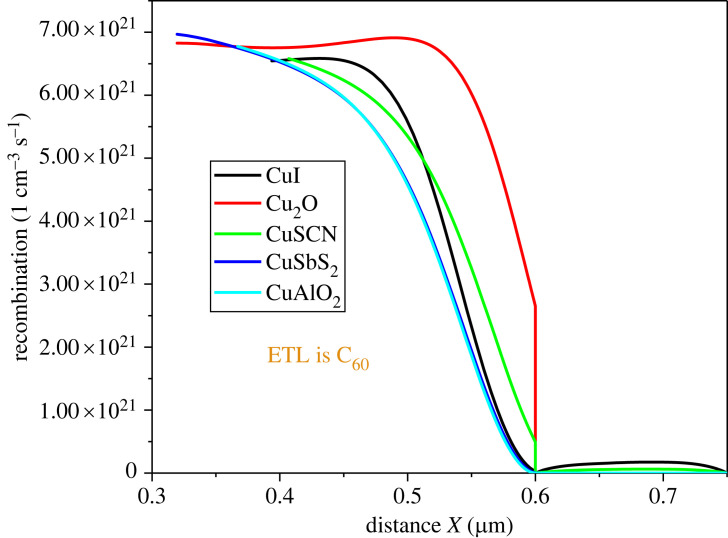
Recombination at perovskite HTL heterojunctions.

**Figure 8.**
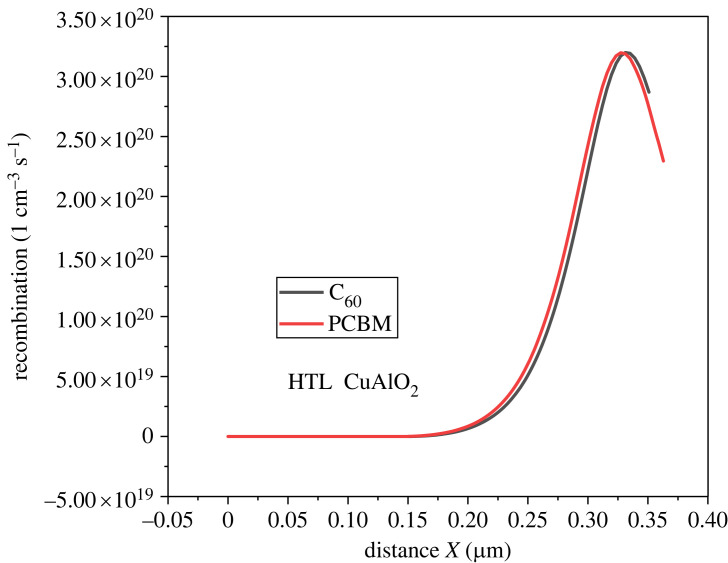
The electric potential at ETL perovskite heterojunction.

Recombination takes place when the electron gets trapped within the material's crystal defects, trap states, or band offsets and completes its carrier's lifetime. This leads to recombination [70]. In perovskite, the recombination occurs at different locations, including the perovskite/electrode interface, within the bulk of the perovskite layer, and at defect centres [71,72]. Figure 7 shows the rate of recombination at the heterojunction of different HTL with MASnI_3_, while figure 8 illustrates the recombination of ETL with the perovskite. CuI, CuSCN, CuSbS_2_ and CuAlO_2_ have low recombination rates at the heterojunction in inverted structures, making them promising candidates for use in perovskite devices as HTL materials. The recombination rate of Cu_2_O is relatively high which makes it less favourable for use. The organic ETLs are good candidates for inverted structures owing to their low recombination rate. This helps in enhancing the overall performance of the device.

## 5. Quantum efficiency

**Figure 9.**
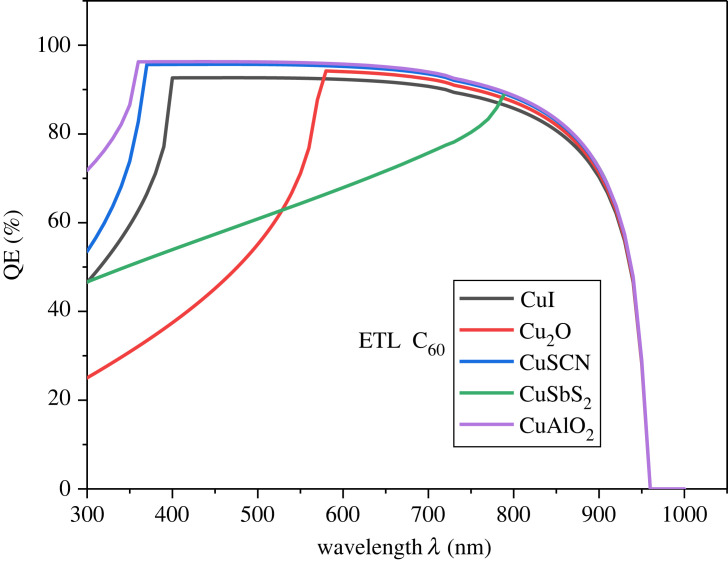
Effect of HTL on QE.

To get the spectral response, the measurement of the QE has been carried out. The QE decreases of the PSC because of recombination [73]. Figure 9 demonstrates the effect of different HTLs on the QE of the PSC.

**Figure 10.**
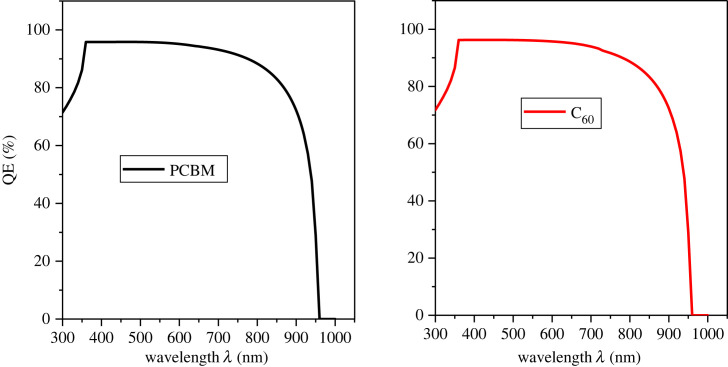
Effect of ETL on QE.

The QE from 300 nm to 400 nm increases up to 90% for CuAlO_2_/MASnI_3_/C_60_, where it goes up to near the infrared region. Generally, good photon-to-charge conversion is observed within the visible range from 300 nm to 700 nm with very much less decrement and almost remains constant. QE falls to zero when the wavelength of the photon is greater than 950 nm as it exceeds the absorption range of the perovskite material. The effect of different ETLs on the QE of the PSC is presented in figure 10. The QE of both ETLs is 90% to 95% for photons of wavelength (300 nm to 850 nm).

### 5.1. J-V characteristic curve

**Figure 11.**
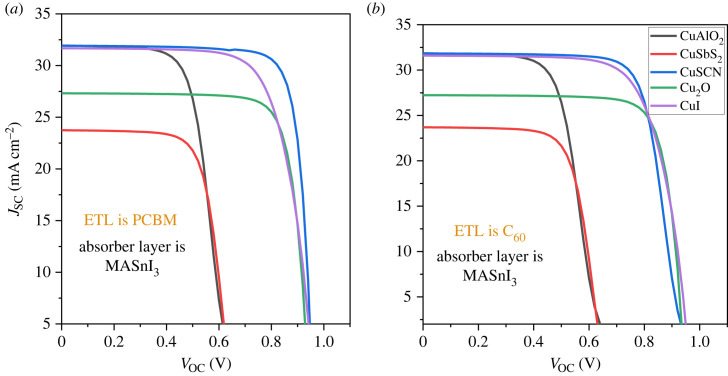
(*a*) J-V curve with PCBM as ETL and (*b*) J-V curve with C_60_ as ETL.

The effect of the different inorganic HTLs and carbon ETLs with Sn-based lead-free PSCs have been studied in detail. Figure 11*a* presents the J-V characteristics curve of p-i-n PSCs with PCBM as ETL. The results show that the structure PCBM/MASnI_3_/CuSCN produces the highest PCE of 22.34%, fill factor (FF) of 73.53%, short circuit current density (*J*_SC_) of 31.83 mA cm^2^ and open circuit voltage (*V*_OC_) of 0.954 V. Figure 11*b* presents the J-V characteristics curve of p-i-n PSCs with C_60_ as ETL. Cu_2_O/PAL/C_60_ and CuI/PAL/C_60_ produce excellent performance with PCE of 20.40% and 21.74%, respectively.

### 5.2. Effect of the active layer thickness

**Figure 12.**
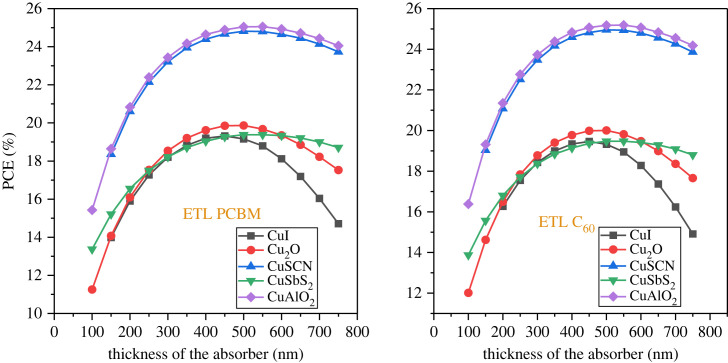
Power conversion efficiency (PCE) versus absorber thickness.

**Figure 13.**
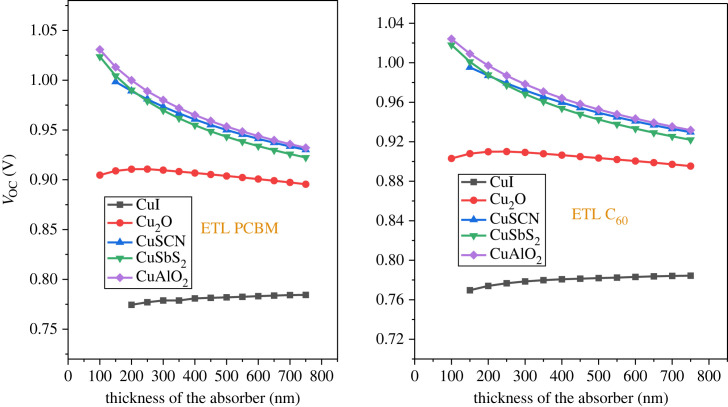
Open circuit voltage (*V*_OC_) versus absorber thicknesses.

**Figure 14.**
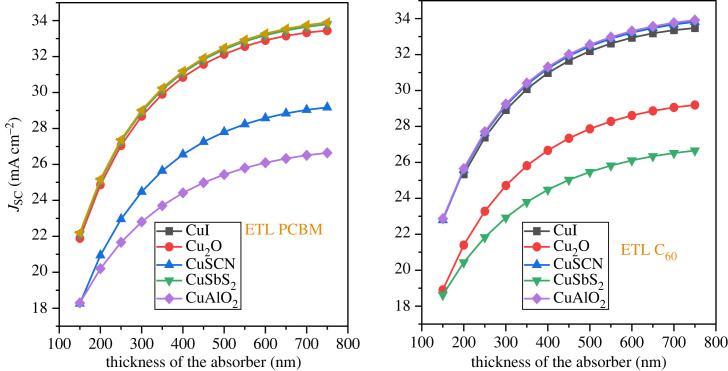
Short circuit current (*J*_SC_) versus active layer thickness.

The optimized absorber layer thickness is fundamental for the high efficiency of the solar cell. The thickness of the active layer is varied from 50 nm up to 750 nm with a 50 nm step while keeping the other parameter of the material constant. Achieving the highest possible number of electron-hole pairs necessitates the determination of an optimal thickness that can effectively absorb the maximum quantity of photons [74]. However, very thick perovskite layers produce low PCE as the thickness of the material exceeds the carrier lifetime [75]. The impact of the absorber thickness on PCE is shown in figure 12. As the thickness increased to 500 nm, all the structures see an increase in PCE, after which saturation occurs. The result is almost the same for both the ETL (PCBM and C_60_)_._ Figures 13 and 14 show that *J*_SC_ increases while *V*_OC_ decreases with an increment in thickness. The decrease of *V*_OC_ with higher thickness is owing to the uplift of the saturation current *I*_o_ which applies more opportunity to e-h pair recombination [7]. The optimized thickness for the structure CuAlO_2_/MASnI_3_/C_60_ results in enhanced performance with PCE of 25.18%, *V*_OC_ of 0.953 V, *J*_SC_ of 32.55 mA cm^2^ and FF of 81.12%. The result of CuSCN/MASnI_3_/C_60_ is almost similar to that of CuAlO_2_.

### 5.3. Effect of inorganic copper-based hole transport material thickness

**Figure 15.**
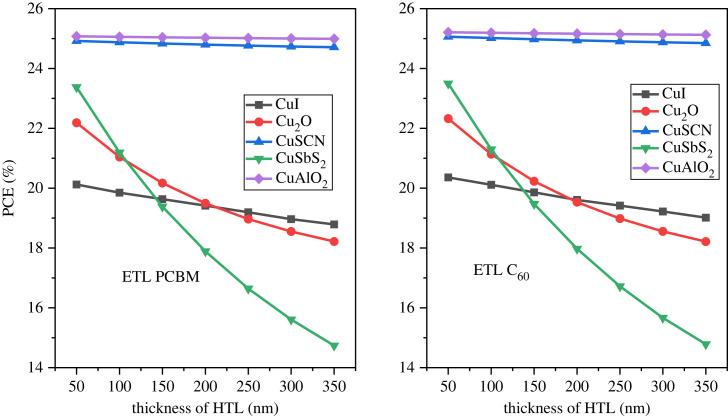
HTL thickness versus PCE.

**Figure 16.**
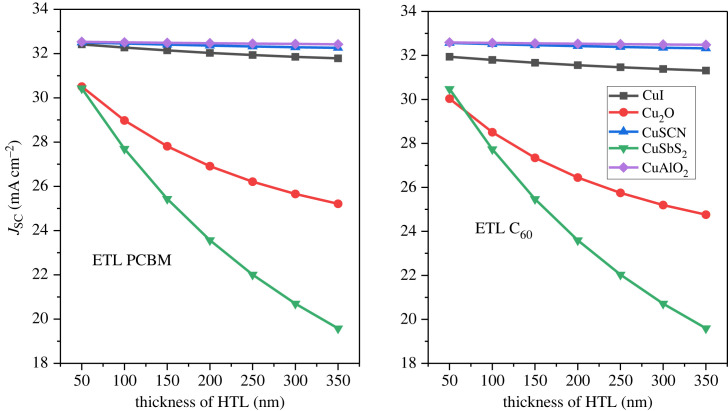
*J*_SC_ versus HTL thickness.

**Figure 17.**
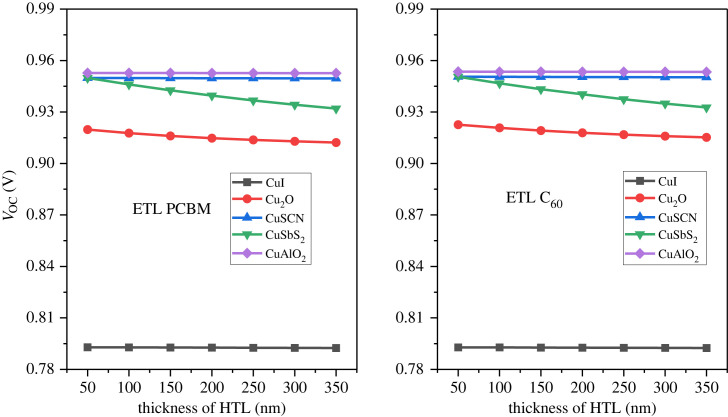
*V*_OC_ versus HTL thickness.

The device efficiency can be further enhanced by using optimized thickness for the inorganic HTL [21]. The HTL is used as the front layer in inverted PSC structures. The optimized value of the active layer was used to calculate the thickness effect of the HTL layers. The thickness of the p-type layer varied from 50 nm to 350 nm with a 50 nm step. Figure 15 represents the HTL thickness effect on the PCE, figure 16 illustrates the thickness effect on *J*_SC_ while figure 17 shows the thickness effect on *V*_OC._

As the thickness increases the PCE, *V*_OC_ and *J*_SC_ decrease. This is because the transparency becomes low and absorption increases of HTL. Further, an increase in HTL thickness also increases the cell's series resistance which reduces performance [76]. The optimal thickness of the HTL is considered 100 nm to provide an adequate buffer and also to minimize the recombination [77,78].

### 5.4. Impact of carbon-based electron transport material thickness

The ETL act as the separating force and capping layer of the cell. The primary and crucial job of the n-type material is to block the hole and extract the electron from the absorber layer [79]. The ETL secondary job is to act as a separation layer between the perovskite and the cathode. A direct contact between the organic perovskite and metallic electrode produces very high resistance and defects [80].

**Figure 18.**
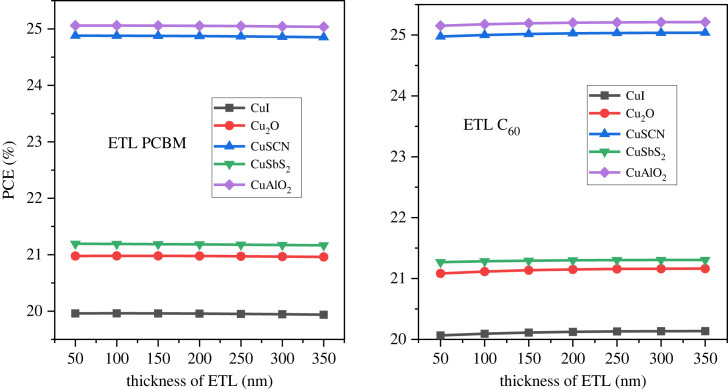
ETL thickness versus PCE.

**Figure 19.**
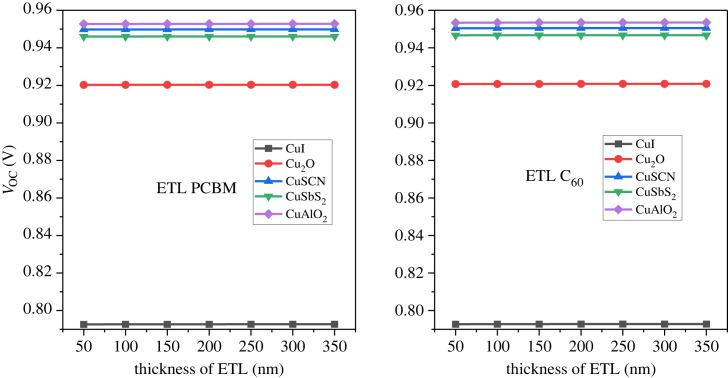
*V*_OC_ versus ETL thickness.

**Figure 20.**
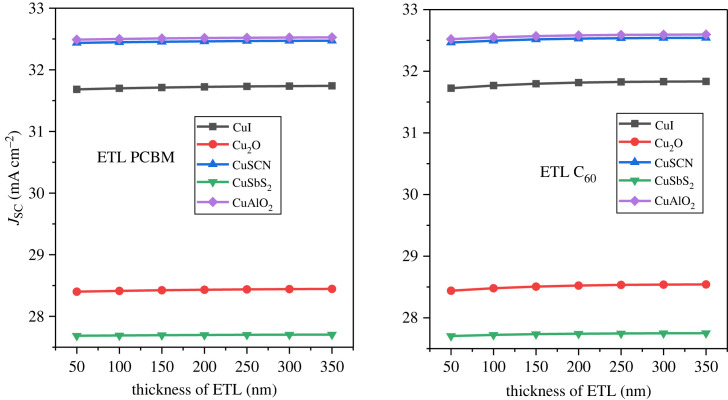
ETL thicknesses versus *J*_SC_.

The analysis of the ETL thickness is performed from a range of 50–350 nm. With increase in thickness of ETL the series resistance on the solar cell increases owing to a rise in defect density of the ETL. This in turn makes it difficult for the electron to reach the cathode and hence recombination occurs. On the other hand, if the thickness of the ETL is reduced too much (below 100 nm) then it becomes too thin to guarantee any adequate separation between the absorber and the electrode. From figures 18–20, it can be observed that there is a very small fluctuation in the PCE, *J*_SC_ and *V*_OC_ with changing thickness. This is owing to the high electric field produced at the hetero-junction by the ETL. The high potential helps the electrons to overcome the traps of the defects and successfully transport them to the cathode. The effect of C_60_ and PCBM thickness is relatively small in the inverted structures because the ETL does not obstruct the light to the active layer [81]. The thickness for all the arrangements is considered as 100 nm for further analysis.

### 5.5. Optimization of doping density (*N_A_*) of absorber layer

The type of doping concentration has an essential effect on the absorber layer. In this study p-type, doping is focussed upon. Increasing the acceptor doping (*N*_A_) concentration enhances the performance of the device by increasing the charge carriers concentration in the absorber [82]. The p-type doping of the absorber layer varied from 10^12^ cm^−3^ to 10^18^ cm^−3^.

**Figure 21.**
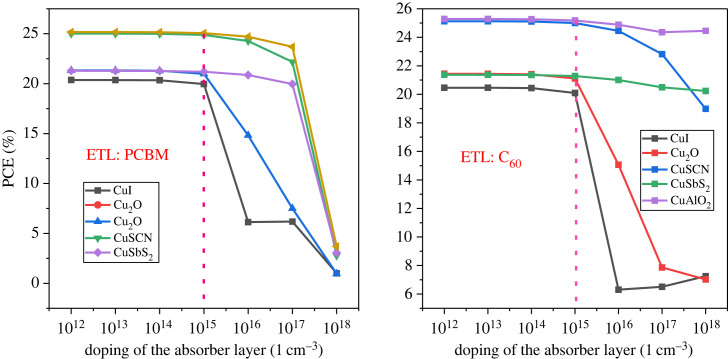
Absorber doping density *N_A_* versus PCE.

**Figure 22.**
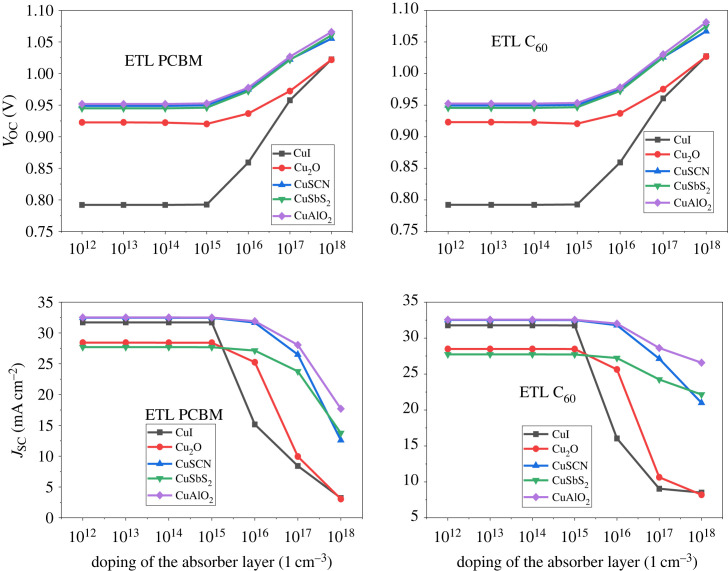
Absorber doping density *N_A_* versus *V*_OC_ and *J*_SC_.

The outcome demonstrated in figures 21 and 22 shows that the result is almost stable until 10^15^ cm^−3^. Further doping drastically reduces the PCE owing to a decrease in *J*_SC_. This occurs because the doping increases auger recombination in the bulk of the absorber [83]. Therefore, the 10^15^ cm^−3^ value for the absorbing doping concentration was selected for further analysis.

### 5.6. Effect of doping density concentrations of charge transport layer

**Figure 23.**
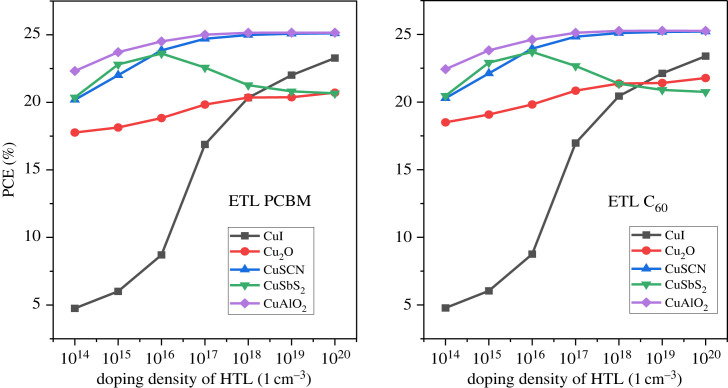
Doping concentration of HTL versus PCE.

**Figure 24.**
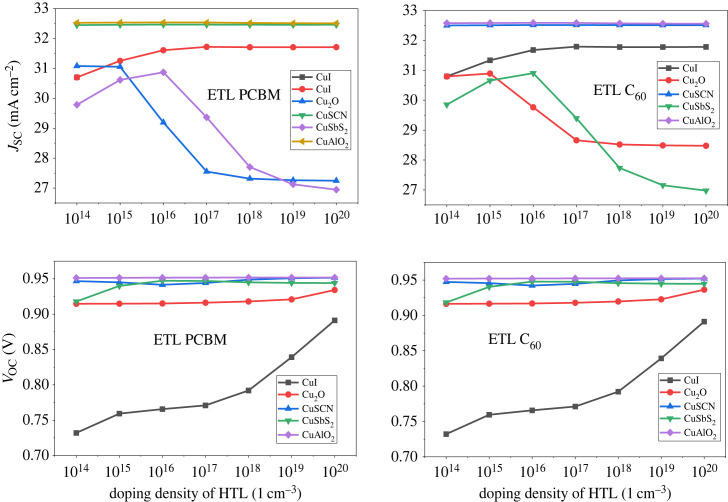
Doping concentration of HTL versus *J*_SC_ and *V*_OC._

In addition to the doping of the active layer, the CTL doping is also important for improving the device result. The doping is performed in two ways, one is minority carrier doping which reduces PCE, *V*_OC_, *J*_SC_, and the other is majority carrier doping which leads to improved efficiency [64]. In this work, the majority carrier doping is focussed upon. For HTL, p-type doping is carried out while for ETL n-type doping [84]. The doping concentrations are varied from 10^14^ cm^−3^ to 10^20^ cm^−3^ for both HTL and ETL. The demonstration of the effect of doping on the HTL is shown in figure 23 which reveals that the PCE reaches the maximum level at 10^20^ cm^−3^. The *J*_SC_ drops while *V*_OC_ rises with increasing doping concentrations of p-type material as shown in figure 24.

**Figure 25.**
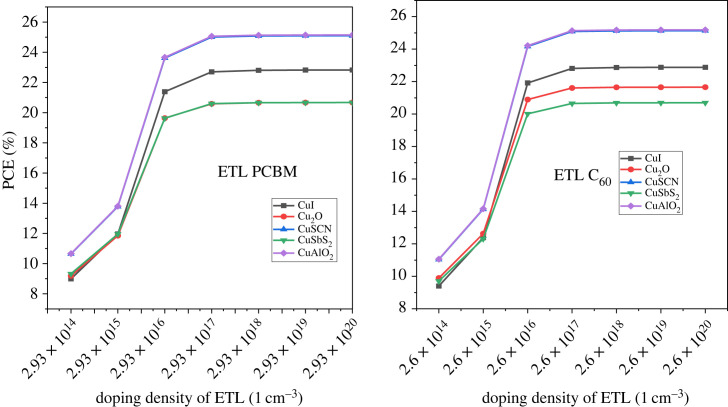
ETL doping density *N_D_* versus PCE.

**Figure 26.**
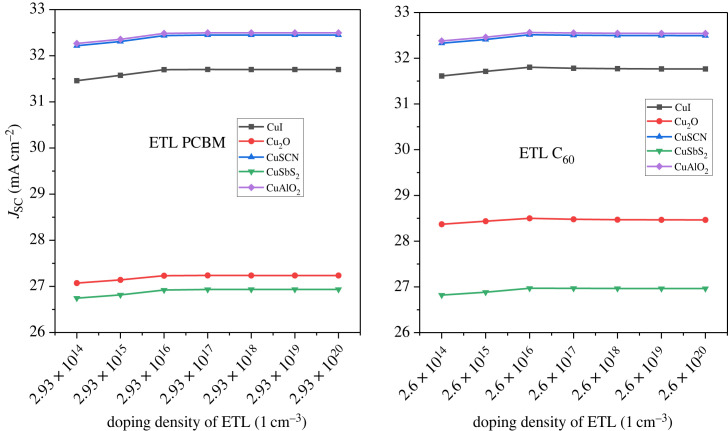
ETL doping density *N_D_* versus *J*_SC_.

**Figure 27.**
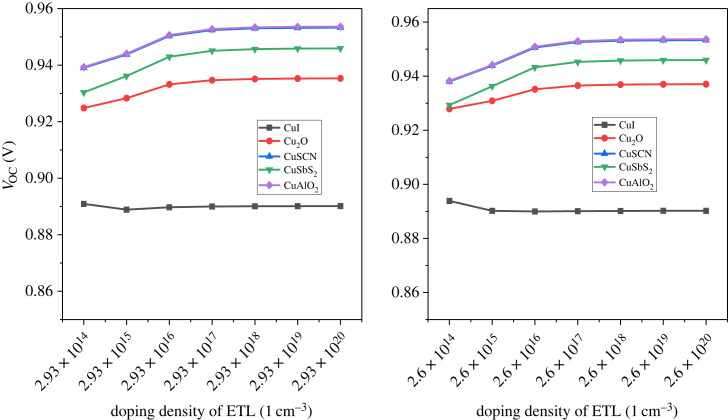
ETL doping density *N_D_* versus *V*_OC_.

For ETL the PCE of PSC increases from doping density 10^14^ cm^−3^ to 10^18^ cm^−3^ as shown in figure 25 and then it saturates. The doping concentration impact on the current density can be observed from the graph in figure 26. In figure 27, the effect of *V*_OC_ is presented. Hence, the ETL doping density is taken at 10^20^ cm^−3^ for efficient performance.

### 5.7. Effect of the defect density (*N_t_*) of the perovskite active layer

**Figure 28.**
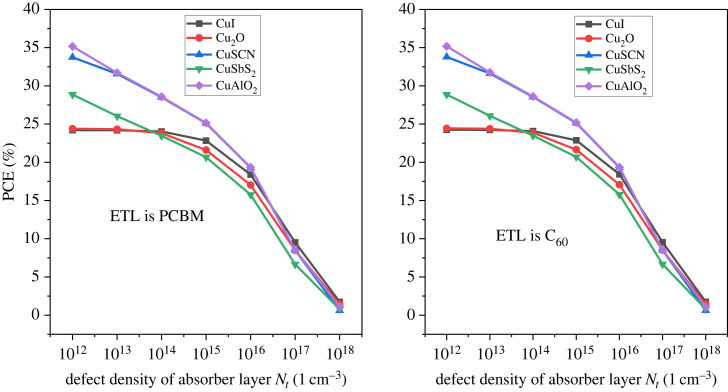
Defect density *N_t_* versus PCE.

The perovskite device performance is affected by the defect density and all its parameter decreases with an increase in it [85]. The carrier generation and recombination that contribute to the device performance are greatly influenced by defect density [86]. When the e-h pair are generated, they have to reach their respective electrodes for collection, but owing to the high defect density of the active layer, the charge carriers complete their carrier life before reaching electrodes. This leads to the poor performance of the device [87]. The defect density *N_t_* is varied from 10^12^ cm^−3^ up to 10^18^ cm^−3^ and their effect is displayed in figure 28.

**Figure 29.**
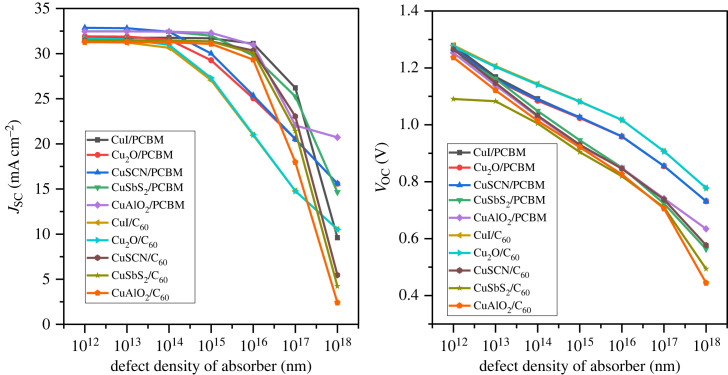
Effect of defect density *N_t_* versus *J*_SC_ and *V*_OC_.

The PCE is stable until 10^15^ cm^−3^ defect density for all configurations but beyond that, the result is drastically changed. The PCE drastically reduce to below 5%. The charge carrier lifetime starts reducing with a rise in defect density [88]. The reported experimental value for the defect density is in the range of 10^14^ cm^−3^ to 10^16^ cm^−3^ in polycrystalline PSCs [89,90]. Figure 29 illustrates the effect of the defect density of the absorber layer on *V*_OC_ and *J*_SC_.

### 5.8. Effect of interface defect

Interface (IF) defect is one of the factors that limit the performance of PSC devices. IF defect refers to the structural and compositional variation at the interfaces between the perovskite absorber layer and the CTL [90,91]. The presence of defects at the interface between ETL/perovskite and perovskite/HTL can lead to the formation of trap states and act as the recombination centre for the charge carrier [92]. This results in the reduction of photocurrent and an increase in the dark current, reducing the overall efficiency of the device [60]. Another effect of this defect is an increase in the series resistance of the device. The influence of the IF defect on the efficiency of the device is investigated through simulation by varying it from 10^10^ cm^−3^ to 10^19^ cm^−3^ for the interface between ETL/perovskite active layer (PAL) and PAL/HTL [7,93].

**Figure 30.**
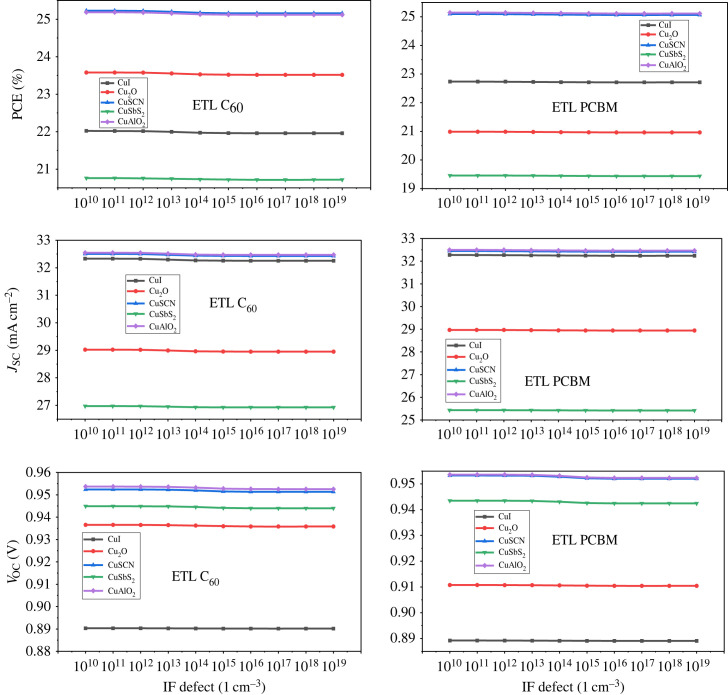
Interface defect effect at ETL/perovskite.

**Figure 31.**
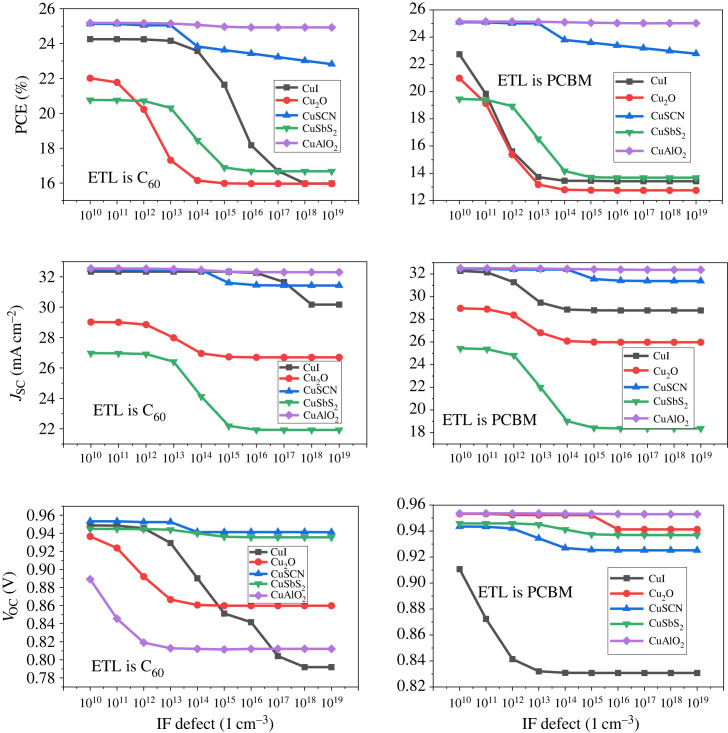
Interface defect effect at perovskite/HTL.

Figure 30 presents the effect of defect at the interface between ETL/perovskite which shows that there is a small effect on the performance of the device when the IF defectincreases when the defect at the other interface (perovskite/HTL) increases there is a sharper decrease in *V*_OC_ and *J*_SC_ owing to the addition of trap levels which results in a significant reduction in PCE shown in figure 31.

### 5.9. Effect of temperature

**Figure 32.**
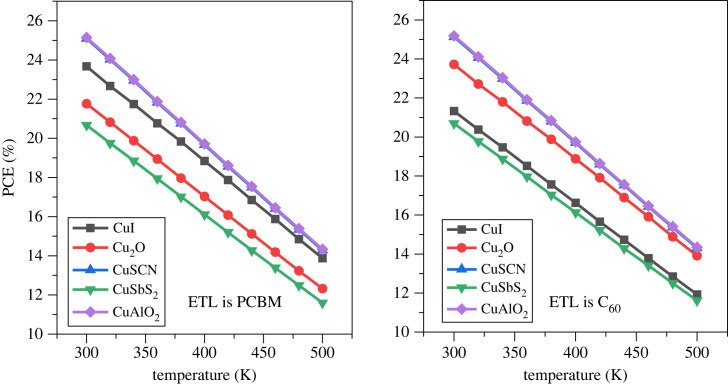
Thermal stability of various hole transport materials with increasing temperature.

**Figure 33.**
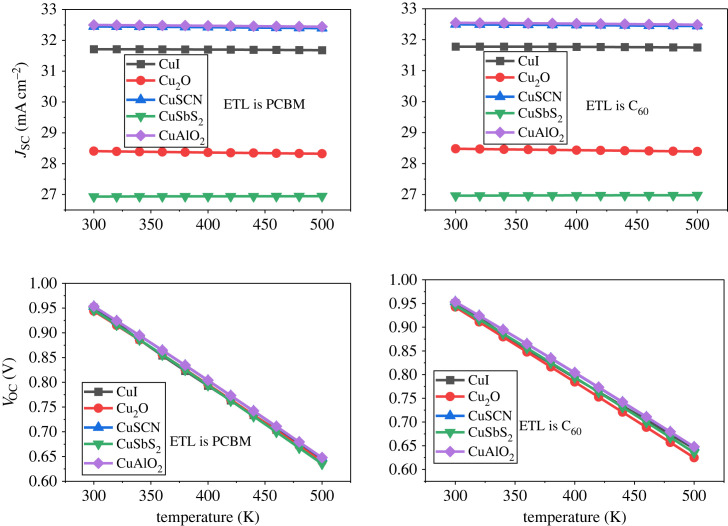
Effect of temperature on *J*_SC_ and *V*_OC_.

The impact of temperature on the performance of the PSC is a crucial factor as it determines the stability of the device [73,94]. The temperature is kept constant at 300 K during the optimization of the parameters of the PSC. To check the thermal stability the temperature is varied from 300 K to 500 K and the influence is shown in figure 32. An increase in temperature drastically reduces the PCE of the PSC. The temperature variation effect on *V*_OC_ and *J*_SC_ can be seen in figure 33 which reveal that current density is stable for all structure, while open circuit voltage is significantly deteriorating when temperature increases [95].

## 6. Metalwork function

**Figure 34.**
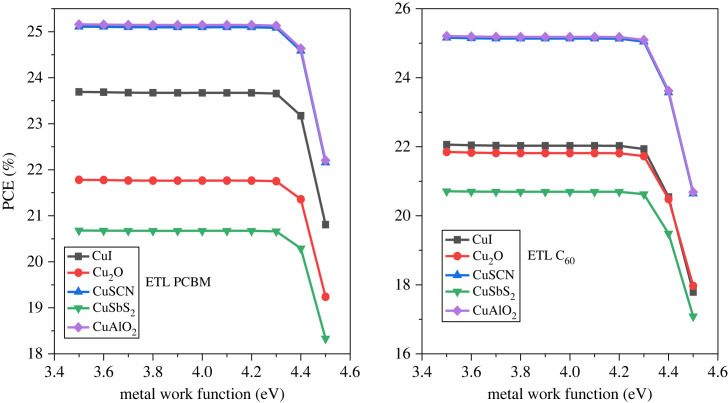
Different metalwork functions affect PCE.

**Figure 35.**
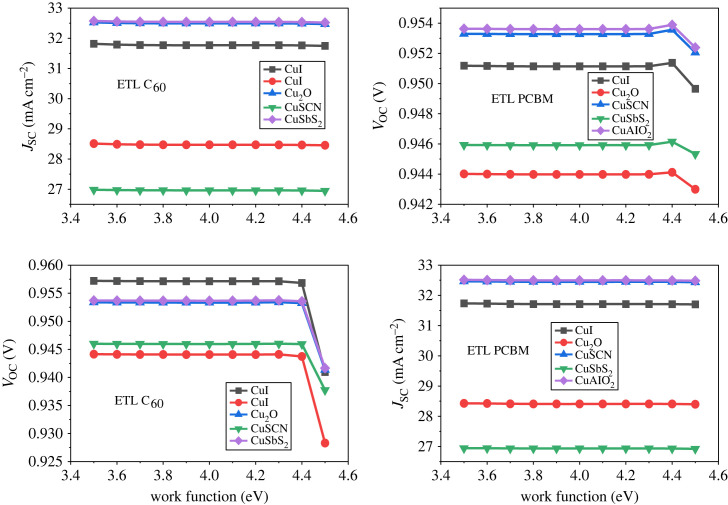
Different work function effects on *J*_SC_ and *V*_OC_.

The work function (WF) of the metal is a crucial factor that influences the operational efficiency of the device. The definition of the WF entails the disparity in energy levels between the Fermi level and the vacuum energy level [96]. A metal WF suitable for the device is essential, therefore the WF effect was studied to know its impact on the PSCs [97]. A high WF is used for the collection of holes in a regular planar structure, while to collect electrons by the cathode relatively low WF is incorporated [98,99]. Therefore, the WF is varied from 3.5 eV to 4.5 eV for an appropriate cathode. The result of the simulation is given in figure 34, which reveals that the power conversion efficiency of PCE is stable for all arrangement of p-i-n inverted perovskite from 3.5 eV to 4.3 eV. Beyond 4.3 eV the parameter such as PCE starts decreasing, which means that the carrier barrier height is in elevation owing to which the recombination rate is high. Figure 35 shows that the current is not affected when WF is increased from 4.3 eV but *V*_OC_ starts falling. As from the literature, the WF of Al is 4.2 eV therefore, it is the best choice for the effective performance of the device.

### 6.1. n-i-p versus p-i-n structure

**Figure 36.**
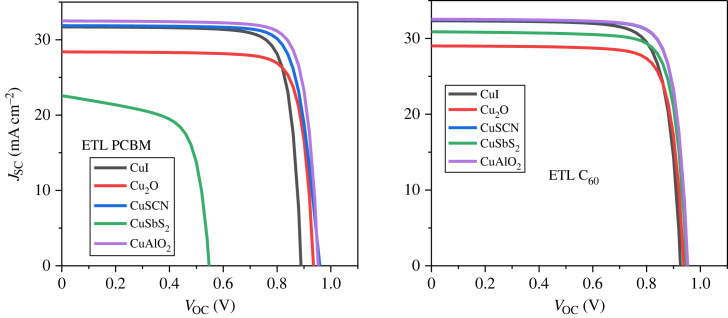
Optimized J-V curve of p-i-n structures.

**Figure 37.**
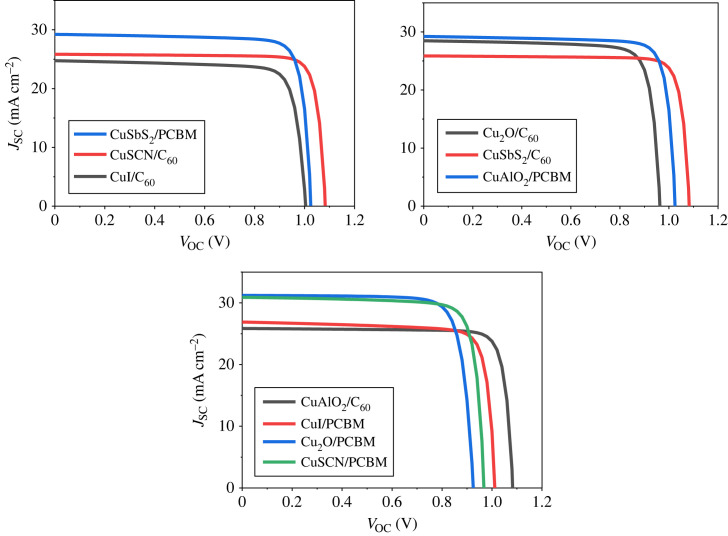
Optimized J-V curve of n-i-p structures.

Outcomes of the enhanced n-i-p and p-i-n PSCs are displayed in tables 3 and 4 for comparison. The performance and stability of the inverted arrangements are better than the regular planar structures [100]. The optimized results of all arrangements are shown in figures 36 and 37 which reveal that the current density curve of the inverted configurations consisting of ETL C_60_ has uniform and stable results as compared to the other structures.

**Table 3.** The output of the optimized regular planar n-i-p structures.

structure arrangement	thickness (nm)	*V*_OC_ (V)	*J*_SC_ (mA cm^−12^)	FF (%)	PCE (%)
FTO/PCBM/MASnI_3_/CuI/Pt	700	1.01	26.72	82.33	22.25
FTO/PCBM/MASnI_3_/Cu_2_O/Pt	700	0.92	31.21	81.44	23.48
FTO/PCBM/MASnI_3_/CuSCN/Pt	750	0.97	30.90	82.29	24.64
FTO/PCBM/MASnI_3_/CuSbS_2_/Pt	750	1.03	29.23	83.64	25.08
FTO/PCBM/MASnI_3_/CuAlO_2_/Pt	750	1.03	29.21	83.64	25.06
FTO/C_60_/MASnI_3_/CuI/Pt	700	1.01	24.81	82.52	20.67
FTO/ C_60_/MASnI_3_/Cu_2_O/Pt	700	0.96	28.26	81.42	22.09
FTO/ C_60_/MASnI_3_/CuSCN/Pt	750	1.08	25.78	85.83	23.96
FTO/ C_60_/MASnI_3_/CuSbS_2_/Pt	750	1.08	25.87	85.84	24.06
FTO/ C_60_/MASnI_3_/CuAlO_2_/Pt	750	1.08	25.85	85.84	24.04

**Table 4.** Result of the optimized inverted perovskite solar cell configurations.

structure arrangement	thickness (nm)	*V*_OC_ (V)	*J*_SC_ (mA cm^–2^)	FF (%)	PCE (%)
ITO/CuI/MASnI_3_/PCBM/Al	500	0.89	31.70	80.90	22.83
ITO/Cu_2_O/MASnI_3_/PCBM/Al	500	0.94	27.24	81.15	20.67
ITO/CuSCN/MASnI_3_/PCBM/Al	500	0.95	32.45	81.13	25.10
ITO/CuSbS_2_/MASnI_3_/PCBM/Al	500	0.95	26.93	81.14	20.67
ITO/CuAlO_2_/MASnI_3_/PCBM/Al	500	0.95	32.50	81.15	25.15
ITO/CuI/MASnI_3_/C_60_/Al	500	0.89	31.77	80.91	22.88
ITO/Cu_2_O/MASnI_3_/ C_60_/Al	500	0.94	28.47	81.18	21.65
ITO/CuSCN/MASnI_3_/ C_60_/Al	500	0.95	32.49	81.13	25.13
ITO/CuSbS_2_/MASnI_3_/ C_60_/Al	500	0.95	26.96	81.14	20.70
ITO/CuAlO_2_/MASnI_3_/ C_60_/Al	500	0.95	32.54	81.15	25.18

## 7. Conclusion

A comprehensive examination has been performed to achieve the best configuration of the perovskite device by using various copper-based p-type materials and carbon-based n-type materials. The inorganic hole transport material has high chemical stability as compared to organic material. The effect of several parameters has been studied such as thickness, doping concentrations and defect density of each layer has been optimized. The optimum thickness for the active layer was around 500 nm for the inverted p-i-n structure. The results of the planar regular n-i-p structure and inverted p-i-n have been compared. It is concluded that the light transparency of HTL as a window layer is high than that of the ETL, therefor the result of the inverted structure was better for all arrangements instead of Cu_2_O. The inorganic HTL has shown good stability with lead-free perovskite using ITO as a transparent conducting oxide. The structure ITO/CuAlO_2_/MASnI_3_/C_60_/Al exhibited the best performance PCE of 25.18%. It is found that the photovoltaic parameters of the device decrease gradually for the back metal contact having a WF above 4.3 eV.

## Data Availability

The datasets generated and analysed during the current study can be accessed on Dryad Digital Repository: https://doi.org/10.5061/dryad.z34tmpgkx [101]. Supplementary material is available online [102].
